# Hygienic-sanitary quality of donated human milk in terms of the donor profile and pumping site

**DOI:** 10.1590/1980-220X-REEUSP-2024-0126en

**Published:** 2025-01-13

**Authors:** Ana Carolina de Prima Souza, Ana Carolina Lavio Rocha, Lucíola Sant’Anna de Castro, Bárbara Tideman Sartorio Camargo, Rosana Rodrigues Figueira Fogliano, Miriam Harumi Tsunemi, Kelly Pereira Coca

**Affiliations:** 1 Universidade Federal de São Paulo, Escola Paulista de Enfermagem, Departamento de Enfermagem na Saúde da Mulher, São Paulo, SP, Brazil.; 2 Universidade Federal de São Paulo, Banco de Leite Humano da SP, Centro de Aleitamento Ana Abrão, Brazil.; 3 Universidade Estadual Paulista Júlio de Mesquita Filho, Instituto de Biociências, Departamento de Biodiversidade e Bioestatística, Botucatu, SP, Brazil.

**Keywords:** Milk, Human, Milk Banks, Breast Milk Expression, Quality Control, Leche Humana, Bancos de Leche Humana, Extracción de Leche Materna, Control de Calidad

## Abstract

**Objective:**

To analyze the factors associated with the hygienic-sanitary quality of donated human milk in terms of the donor profile and pumping site.

**Method:**

Cross-sectional study with retrospective data collection of records of human milk samples donated to a Human Milk Bank in São Paulo, Brazil, from 2014 to 2019. Characteristics of human milk donors, pumping site, and hygienic-sanitary quality were analyzed based on the Standards of the Brazilian Human Milk Bank Network.

**Results:**

In the hygienic-sanitary assessment, a greater presence of contamination, high acidity, and microbiological alteration were found when human milk was pumped at home and when it was donated mature. The human milk from non-exclusive donors, especially those > 30 years old, presented greater microbiological contamination and higher acidity when compared to that from exclusive donors (p < 0.05).

**Conclusion:**

Hygienic-sanitary quality impairment of donated human milk occurred when the collection was performed at home, especially among non-exclusive donors. Effective guidance and indirect supervision of donors who perform home pumping can contribute to better use of donated human milk.

## RESUMEN

**Objetivo:**

Analizar los factores asociados a la calidad higiénico-sanitaria de la leche humana donada en función del perfil del donante y lugar de extracción.

**Método:**

Estudio transversal con recolección de datos retrospectivo de registros de muestras de leche humana donadas a un Banco de Leche Humana en São Paulo, Brasil, de 2014 a 2019. Se analizaron las características de las donantes de leche humana, lugar de extracción y calidad higiénico-sanitaria desde la perspectiva de las Normas de la Red de Bancos de Leche Humana en Brasil.

**Resultados:**

En la evaluación higiénico-sanitaria se identificó mayor presencia de contaminación, alta acidez y alteraciones microbiológicas al extraerse en casa y al donar leche humana madura. Donantes no exclusivos, especialmente aquellos entre mujeres > 30 años, presentaron mayor contaminación microbiológica y mayor acidez en la leche humana donada en comparación con la exclusiva (p < 0,05).

**Conclusión:**

El deterioro de la calidad higiénico-sanitaria de la leche humana donada sucedió cuando la recogida se realizó en casa, especialmente entre donantes no exclusivas. La orientación eficaz y la supervisión indirecta de las donantes que realizaron la extracción en casa pueden contribuir a un mejor uso de la leche humana donada.

## INTRODUCTION

Human milk (HM) is considered the most complete food to be offered to newborns, as it has nutritional and immunological properties to protect the child’s gastrointestinal and respiratory systems^(1)^. The specificity of HM is complex, capable of meeting the child’s numerous needs, either related to the child’s age or the different stages of development and, in particular, to variations of gestational age at birth, such as premature babies^(2,3)^.

Brazil, recognized for its breastfeeding protection programs, is considered a world reference in Human Milk Banks (HMB)^(4)^ and promotes different strategies to protect and support breastfeeding^(5)^ to ensure the benefits of this practice^(6)^. Among them, the collection of donated HM for infants admitted to Neonatal Intensive Care Units (NICU) who do not have their respective mothers’ milk^(7)^ stands out. Because mother-to-child HM is recommended as a priority, women with premature newborns are advised to express their milk until they can breastfeed their baby directly^(8,9)^. Therefore, throughout the infant’s hospitalization period, the Human Milk Collection Station (HMCS) is the support unit for carrying out the in-hospital HM pumping^(10)^.

The breastfeeding protection policy, guaranteed by the Brazilian Network of Human Milk Banks (r-BLH), ensures the quality of HM from pumping to distribution through its well-established hygiene and health control standards and routines^(10,11)^. HM is pumped in the HMCSs throughout the period the woman remains hospitalized. After the postpartum woman is discharged from hospital, HM pumping is also done at home, which allows the mother’s own HM to be available and the maintenance of her milk production, to meet the infant’s needs and in an attempt to maintain exclusive breastfeeding (EBF) directly from the breast^(12)^.

Among the hygienic and sanitary recommendations for milk pumping, there are: choosing a clean and calm environment, removing ornaments and tying up hair, wearing the attire (a cap and mask), sanitizing her hands, massaging her breasts in circular motions, cleaning the breasts using gauze and clean water or saline solution, and starting collection after discarding the first jets from hand expression, collecting the HM in a glass container with a sterilized plastic lid. If using a breast pump, sterilization of the utensils is also recommended^(10,11)^. Immediately after pumping, HM should be labeled and stored in a frozen state at -20°C, in a freezer, in an attempt to preserve nutrients and prevent the proliferation of pathogenic microorganisms^(13)^.

Regarding distribution, Brazil determines that HM should only be administered raw, that is, without going through the pasteurization process, when expressed at the bedside and/or in the HMCSs from mother to child, under the supervision of a health professional^(13)^. In contrast, HM collected at home must necessarily undergo quality control and then be pasteurized before distribution, regardless of whether it is HM from mother to child^(10,14)^, since there is a risk of secondary contamination during pumping, compromising the quality and safety of the HM to be provided^(10,14,15)^.

Despite this recommendation in Brazil, other countries allow the provision of raw HM from mother to child, regardless of the pumping place^(16)^. Studies comparing risks and benefits between HM pumping sites in terms of hygienic-sanitary quality are scarce^(17,18)^. A study carried out in 2014 in Australia compared HM samples collected at the HMB and at home, and did not identify any significant difference regarding the location of HM collection, presenting an approval rate of 98% and 94% for samples collected at the HMB and at home, respectively^(19)^.

This way, the analysis of the hygienic-sanitary quality of the HM collected at home, as well as its comparison with the HM collected in the HMCSs, can support the practices already established and contribute to updating the current standards.

Therefore, this study aimed at analyzing the factors associated with the hygienic-sanitary quality of donated HM as for the donor profile and pumping site.

## METHOD

### Study Design And Local

This is a cross-sectional study with retrospective data collection of records of HM samples donated to the HMB of a University Hospital, which was part of the project entitled “Evaluation of the indicators of a HMB”.

The HMB of the Universidade Federal de São Paulo (UNIFESP) is located in the Brazilian Southeast region and carries out around 800 home visits/year to collect HM, obtaining approximately 600 liters/year. The HMCSs distribute around 90 liters of HM/year to an average of 260 infants/year. Most of the infants who receive donated HM are premature and admitted to the Neonatal ICU of University Hospital/ Hospital São Paulo (HU/HSP).

### Population and Selection Criteria

The sample consisted of all HM donation records from 2014 to 2019. The HM records of donors considered suitable for donation were included, according to the criteria established by the rBLH and the Brazilian Health Regulatory Agency (ANVISA)^(10)^. The exclusion criterion established was the registration of donated HM with incomplete data related to the date of birth of the donors and infants, which hinders the analysis of the donated HM and, consequently, prevents the achievement of the study objective proposed.

### Data Collection

Data collection was carried out by two researchers trained to obtain the variables selected for the study from the donors’ electronic medical records and from the HMB’s monthly production data spreadsheet, from August 2019 to August 2020.

Variables were collected to characterize the donor: maternal age (≤ 29 e > 30 years; subdivision of the reproductive range of the Brazilian Institute of Geography and Statistics - IBGE); postpartum period of donation (≤ 1 month and > 1 month); and variables related to donated HM: HM phase (colostrum, transition, and mature) and energy value (hypocaloric: < 500 kcal/l, normocaloric: 500–700 kcal/l, and hypercaloric: > 700 kcal/l). The outcome variables established were: type of donation (exclusive or non-exclusive), pumping site (HMCSs and home) and hygienic-sanitary quality of the donated HM. The hygienic-sanitary quality was verified for: 1) presence of physical contamination, 2) off flavor, 3) Dornic acidity, and 4) microbiological analysis. Therefore, quality was considered compromised when there was presence of physical contamination, altered odor, higher acidity, and microbiological contamination^(10)^.

Physical contamination was defined considering personal cleanliness (presence of body and head hair and skin inside the HM container) and/or environment (presence of fabric lint, insects, and food inside the HM container), being categorized according to its origin.

The off flavor test was determined by evaluating the flavor, prior to the pasteurization process, in which the presence of the smell of coconut soap, fish, medicine, chlorine, plastic, and/or rubber characterizes the HM as unsuitable for consumption.

Determination of acidity in HM was measured through Dornic Acidity (AD), in which a value ≤8°D is considered adequate, using a Dornic acidimeter with a fine tip, graduated in 0.01 mL, a factored standard solution of 0.1N sodium hydroxide, and a neutralized 1% hydroalcoholic phenolphthalein indicator solution in 95°GL alcohol (ninety-five degrees Gay-Lussac).

Microbiological analysis was performed by inoculating four aliquots of 1 mL each of pasteurized HM, pipetted independently and inserted into tubes with 10 mL of brilliant green bile broth (BGBL), at 50 g/L (5% w/v), with Durham tubes inside. After inoculation and incubation at 36 ± 1°C, the presence of gas inside the Durham tube characterizes a positive result, with the acceptable parameter being negative or absent.

To compare the types of donation, data from women who voluntarily expressed their intention to donate their HM were considered, who were called non-exclusive donors, while those with hospitalized infants were called exclusive donors. Regarding the place where the donated HM was pumped, since exclusive donors pumped their HM both at the HMCSs and at home, these two places were considered when comparing the hygienic-sanitary quality of the HM among the donors.

The HM donated to the UNIFESP HMB is transported frozen, under a cold chain, in an isothermal container with recyclable ice, maintaining a maximum temperature of -1°C, according to the recommendations of the Brazilian rHMB^(7)^. Transportation is carried out by a trained healthcare professional. In the case of exclusive donors, the HM is transported by the postpartum woman or a family member, after receiving instructions for appropriate transport, which includes keeping the HM frozen in an appropriate container. Upon arrival at the institution, all HM received is checked to ensure that it is suitable for processing, which includes checking that the vial is suitable, within the expiration date, and that it is frozen correctly. If the HM delivered by the postpartum woman presents inadequacies, it will be discarded at reception.

The information was entered into a database built in Microsoft Office Excel 2016®. The records were double-checked to confirm all the information obtained.

### Data Analysis

In the description of the data, qualitative variables and their respective relative (%) and absolute (n) frequencies were used, and for the quantitative variables, mean and standard deviation (SD) were used. To compare the variables, type of donor, pumping site, and hygienic-sanitary quality of the HM, the chi-square or Fisher’s exact test was used, considering the level of statistical significance α equal to 5% (p < 0.05). The software Stata/SE 14.0 (Texas, USA) was used for data analysis.

### Ethical Aspects

The study was approved by the Research Ethics Committee of the Universidade Federal de São Paulo (project no. 0712/2018, opinion no. 2.755.431), in accordance with the Brazilian Research Ethics Committee (CONEP) resolution 466 of 2012. As this was a retrospective study using data from medical records, the use of the Free Informed Consent was waived.

## RESULTS

A total of 14,824 HM samples from 986 donors were analyzed. The majority of postpartum women were over 30 years old (75.2%; 30.7 years; SD = 6.6) and, at the time of HM donation, were more than one month postpartum (77.7%). The donated HM samples were mostly classified as mature (89.2%) and normocaloric (54.2%). The majority of samples were from non-exclusive donors (66.4%), and most pumped at home(77.3%) (Table 1).

**Table 1:** Characterization of donors and human milk samples extracted, according to the type of donor – Sao Paulo, SP, Brazil, 2014–2019.

Characterization variables	Non-exclusive donors n (%)	Exclusive donors n (%)	P
**Donors**			
Maternal age (n = 986)			0.867
≤ 29 years old	2,441 (24.8)	1,229 (24.7)	
> 30 years	7,402 (75.2)	3,752 (75.3)	
*Postpartum period* (n = 14,824)			0.818
≤ 1 month	2,198 (22.3)	1,104 (22.2)	
> 1 month	7645 (77.7)	3,877 (77.8)	
**HM donation**			
Pumping site (n = 14,824)			**<0.001**
**At home**	9,843 (100)	1,617 (32.5)	
HMCSs	0 (0)	3,364 (67.5)	
*HM phase* (n = 14,816)			**<0.001**
Colostrum	6 (0.1)	137 (2.8)	
Transition	191 (1.9)	1,271 (25.5)	
Mature	9,646 (98.0)	3,565 (71.7)	
*Caloric value* (n = 11,210)			**0.001**
Hypocaloric	1,734 (23.5)	868 (22.7)	
Normocaloric	3,905 (52.9)	2,162 (56.4)	
Hypercaloric	1,739 (23.6)	802 (20.9)	

The presence of physical contamination was found in 22% (n = 1,095/4,981) of the HM samples from exclusive donation, while for non-exclusive donation this number was 25% (n = 2,459/9,843), with no statistically significant difference. And when assessed by type, physical contamination classified as environmental was more prevalent in non-exclusive donation samples (p = 0.039). Regarding the off flavor test, the minority, 0.1% (n = 18/14,824), of the HM samples presented alterations, and those with altered odors were extracted at home, by non-exclusive donors (p < 0.001). Regarding the acidity titration (AD) test, the majority of HM, 98.9% (n = 12,868/13,018), was adequate. Regarding the microbiological analysis, among the altered samples, 76.3% (n = 748/981) were from non-exclusive donors (p < 0.001).

Table 2 shows that, among the factors associated with hygienic-sanitary changes in the pumped HM, being more than 1 month postpartum and having pumped at home were related to the presence of physical contamination (p < 0.001 and p = 0.048, respectively). Furthermore, non-exclusive donation (p < 0.001) of mature HM one month postpartum (p = 0.024) and pumping of HM at home (p < 0.001) were related to changes in acidity. Non-exclusive donation, especially among women over 30 years of age and those who performed the extraction at home, was associated with changes in the results of microbiological analysis (p < 0.001, p = 0.004 and p < 0.001, respectively).

**Table 2:** Factors associated with the hygienic-sanitary control of extracted human milk – Sao Paulo, SP, Brazil, 2014–2019.

	Physical contamination			Off flavor			Acidity			Microbiological analysis*		
	Absence n (%)	Presence n (%)	p	Normal n (%)	Changed n (%)	p	Normal n (%)	Changed n (%)	p	Normal n (%)	Changed n (%)	p
**Donor characteristic**
Non-exclusive	7,584 (66.1)	2,259 (67.4)		9,829 (66.4)	14 (77.8)		8,462 (65.8)	137 (91.3)		6,691 (64.9)	748 (76.3)	
Exclusive	3,886 (33.9)	1,095 (32.6)	0.184	4,977 (33.6)	4 (22.2)	0.306	4,406 (34.2)	13 (8.7)	**<0.001**	3,617 (35.1)	233 (23.7)	**<0.001**
**Breastfeeding woman age**
≤ 29 years old	2,866 (25)	804 (23.9)		3,666 (24.8)	4 (22.2)		3,180 (24.7)	40 (26.7)		2,537 (24.6)	282 (28.7)	
> 30 years	8,604 (75)	2,550 (76)	0.231	11,140 (75.2)	14 (77.8)	0.803	9,688 (75.3)	110 (73.3)	0.581	7,771 (75.4)	699 (71.3)	**0.004**
**Postpartum period**
≤ 1 month	2,539 (22.1)	763 (22.8)		3,299 (22.3)	3 (16.7)		2,869 (22.3)	45 (30)		2,271 (22)	222 (22.6)	
> 1 month	8,931 (77.9)	2,591 (77.2)	0.453	11,507 (77.7)	15 (83.3)	0.567	9,999 (77.7)	105 (70)	**0.024**	8,037 (78)	759 (77.4)	0.666
**Pumping site**
Domicile	8,713 (76)	2,747 (81.9)		11,444 (77.3)	16 (88.9)		9,812 (76.3)	142 (94.7)		7,765 (75.3)	805 (82.1)	
HMCSs	2,757 (24)	607 (18.1)	**<0.001**	3,362 (22.7)	2 (11.1)	0.240	3,056 (23.7)	8 (5.3)	**<0.001**	2,543 (24.7)	176 (17.9)	**<0.001**

*n = 11,289.

## DISCUSSION

HM pumped at home, mainly by non-exclusive donors over 30 years of age and within 30 days or more postpartum, was associated with a higher prevalence of HM non-compliance, related to insufficient hygienic-sanitary care at the time of HM pumping due to the presence of physical contamination, increased acidity, and changes in microbiological factors in the donated HM. This is the first study to demonstrate the possible risks of using raw HM collected at home, reinforcing the importance of pasteurization.

Despite the widespread recommendation of using raw HM from mother to child, given its importance and immunological benefits^(2)^, there is a need to ensure the food safety of the HM provided to premature newborns^(20)^, given their characteristics and mortality rate^(21)^. The present study showed that HM expressed at home has a higher soiling rate (p < 0.001), which is in line with the Brazilian rHMB recommendation to analyze the quality of all HM pumped at home^(20)^.

Physical contamination occurs mainly due to contact with the external environment and is often associated with carelessness during the pumping technique such as: lack of hand and breast hygiene, wearing loose hair, handling objects while pumping, exposing the container to the environment, and inadequate sterilization of the container^(10,22)^. In the present study, it was observed that most of the physically contaminated samples were related to personal hygiene and environmental care during collection. A study evaluating the impact of measures adopted to reduce the amount of inappropriate HM, through an educational tool with a step-by-step guide to the HM pumping technique and the causes of HM disposal, showed a reduction in contamination from 24% to 10.5%, reinforcing the importance of constant guidance to maintain quality over time^(23)^.

In addition to the risk of contamination by physical contamination during pumping, HM has a great capacity to absorb volatile substances. The detection of non-conforming sensory characteristics, when compared to the original aroma of the HM, through the perception of smell used as a way of classifying the HM as suitable or not for consumption, is a fast and efficient method^(10)^. In the present study, the rate of odor change was identified only among samples that were expressed at home and by non-exclusive donors, although minimal. A study observed a direct relationship between odor modification and microbiological contamination of HM due to inadequate handling and/or storage, such as contamination of HM by lipolytic microorganisms associated with the odor of coconut soap^(24)^. The recommendation to express HM in an environment free from odors, such as perfume and spices, ensures the quality of the product and reinforces the need to protect the extracted HM^(24)^.

Another parameter that classifies and selects HM is Dornic acidity. The low rate of acidity change in HM (1.1%) found in the present study corroborates the study carried out in the city of Uberlândia^(25)^. Conversely, when compared with another study held in Spain, which identified a 12% AD in HM^(26)^, the prevalence of acidity changes above recommended values was significantly higher when pumping was performed at home (p < 0.001). The original acidity of HM varies between 1.0 and 4.0°D and, in an environment favorable to biological growth, the acidity increases through the production of acid. This process of increasing acidity in HM is almost always associated with the actions of microorganisms, which reduces the nutritional and immunological value and makes HM unfit for consumption^(13)^. In addition, other factors that influence acidity variation are: inadequate handling of HM, storage in an inappropriate or incorrectly sterilized container, and temperature fluctuations, either due to delays in storing HM or problems related to the refrigerator^(10)^.

These findings demonstrate the importance of constant guidance and monitoring of the HM donor, to reduce the receipt of donated HM that is unfit for consumption^(27)^. The importance of reducing the donation of HM with altered acidity is highlighted through effective guidance of donors regarding care in handling the sterilized containers made available for HM collection, from immediate freezing to pumping and minimal handling after storage, as well as correct transportation to the HMB^(10)^.

As for microbiological analysis, this is the last hygienic-sanitary quality parameter to be evaluated, which identifies the retention of pathogens due to excessive or faulty handling. This is performed after heat treatment of the donated HM and aims to inactivate microorganisms such as *Staphylococcus aureus, S. epidermidis, Enterobacteriaceae,* fungi, and yeasts that can be found in HM^(10,22,28)^. The microbiological contamination rate of 8.7% was similar to a study conducted at a North American HMB^(28)^. The contamination identified in the present study was higher among women over 30 years old (p = 0.004), in non-exclusive HM donation (p < 0.001) and which was pumped at home (p < 0.001), unlike the study carried out in Spain, in which contamination was higher among exclusive donors^(29)^. These findings reinforce the need for rigorous quality control of donated HM pumped at home^(30)^, as well as the adequate preparation of nursing mothers who wish to donate.

Among the limitations of this study, the use of retrospective data and a cross-sectional analysis may limit the inference of the results, in addition to the smaller number of exclusive donations compared to non-exclusive ones. In contrast, the findings are important to strengthen the current Brazilian rHMB recommendations regarding the importance of quality control of donated HM.

## CONCLUSION

Hygienic-sanitary quality impairment of donated HM occurred when the collection was performed at home, especially among non-exclusive donors.

The finding supports the recommendation regarding the processing of HM from exclusive donors, when expressed at home, and reinforces the importance of health teams working in HMBs and HMCSs establishing a routine of individualized guidelines for better use of donated HM, to guarantee the hygienic-sanitary quality of the HM distributed to premature infants.
