# Supplementary material for: Hygienic-sanitary quality of donated human milk in terms of the donor profile and pumping site
Source: Rev Esc Enferm USP. 2025 Jan 13;58:e20240126. doi: 10.1590/1980-220X-REEUSP-2024-0126en (PMC11745413; doi:10.1590/1980-220X-REEUSP-2024-0126en)
Supplement: Translation

# Translation: Qualidade higiênico-sanitária do leite humano doado quanto ao perfil da doadora e local de extração

**DOI:** 10.1590/1980-220X-REEUSP-2024-0126pt

**Authors:** Ana Carolina de Prima Souza, Ana Carolina Lavio Rocha, Lucíola Sant’Anna de Castro, Bárbara Tideman Sartorio Camargo, Rosana Rodrigues Figueira Fogliano, Miriam Harumi Tsunemi, Kelly Pereira Coca

**Affiliations:** 1 Universidade Federal de São Paulo, Escola Paulista de Enfermagem, Departamento de Enfermagem na Saúde da Mulher, São Paulo, SP, Brazil.; 2 Universidade Federal de São Paulo, Banco de Leite Humano da SP, Centro de Aleitamento Ana Abrão, Brazil.; 3 Universidade Estadual Paulista Júlio de Mesquita Filho, Instituto de Biociências, Departamento de Biodiversidade e Bioestatística, Botucatu, SP, Brazil.

**Keywords:** Leite Humano, Bancos de Leite Humano, Extração de Leite, Controle de Qualidade

## Abstract

**Objetivo:**

Analisar os fatores associados à qualidade higiênico-sanitária do leite humano doado quanto ao perfil da doadora e local da extração.

**Método:**

Estudo transversal com coleta de dados retrospectiva de registros de amostras de leite humano doado para um Banco de Leite Humano em São Paulo, Brasil, no período de 2014 a 2019. Foram analisadas características das doadoras de leite humano, local da extração e qualidade higiênico-sanitária na perspectiva das Normas da Rede de Banco de Leite Humano no Brasil.

**Resultados:**

Na avaliação higiênico-sanitária foram identificadas maior presença de contaminação, acidez elevada e alteração microbiológica no leite extraído em domicílio e no leite humano maduro. O leite humano de doadoras não-exclusivas, especialmente as > 30 anos, apresentou maior contaminação microbiológica e acidez mais elevada quando comparado ao das doadoras exclusivas (p < 0,05).

**Conclusão:**

O comprometimento da qualidade higiênico-sanitária do leite humano doado deu-se quando a coleta foi realizada no domicílio, especialmente entre doadoras não-exclusivas. A orientação efetiva e supervisão indireta às doadoras que realizaram a extração no domicílio pode contribuir para o melhor aproveitamento do leite humano doado.

## INTRODUÇÃO

O leite humano (LH) é considerado o alimento mais completo a ser oferecido ao recém-nascido, por possuir propriedades nutritivas e imunológicas para a proteção do sistema gastrointestinal e respiratório da criança^(1)^. A especificidade do LH é complexa, capaz de atender às inúmeras necessidades da criança, sejam elas relacionadas à idade em que a criança se encontra ou nas diferentes fases de desenvolvimento e, em especial, às variações da idade gestacional de nascimento, como as prematuras^(2,3)^.

O Brasil, reconhecido pelos seus programas de proteção à amamentação, é considerado referência mundial em Bancos de Leite Humano (BLH)^(4)^ e promove diferentes estratégias de proteção e apoio ao aleitamento materno^(5)^ para assegurar os benefícios dessa prática^(6)^. Dentre elas, destaca-se a captação de LH doado para as crianças internadas nas Unidades de Terapia Intensiva Neonatais (UTIN) que não têm o leite de suas respectivas mães^(7)^. Por recomendar prioritariamente o LH de mãe para filho, as puérperas com recém-nascidos prematuros são orientadas a extrair seu leite até que possam amamentar seu filho diretamente na mama^(8,9)^. Para tanto, durante todo o período de internação da criança, o Posto de Coleta de Leite Humano (PCLH) é a unidade de apoio para realizar a extração do LH intra-hospitalar^(10)^.

A política de proteção ao aleitamento materno, garantida pela Rede Brasileira de Bancos de Leite Humano (r-BLH), assegura a qualidade do LH desde a extração até sua distribuição por meio de suas normas e rotinas de controle higiênico-sanitário bem estabelecidas^(10,11)^. O LH é extraído no PCLH durante todo o período em que a mulher se encontra internada. Após a alta hospitalar da puérpera, a extração do LH passa a ser feita, também, em seu domicílio, o que possibilita a disponibilidade de LH da própria mãe e da manutenção de sua produção láctea, para atender às necessidades da criança e na tentativa de manter o aleitamento materno exclusivo (AME) diretamente na mama^(12)^.

Dentre as recomendações higiênico-sanitárias para a extração láctea, têm-se: escolher um ambiente limpo e calmo, retirar adornos e prender o cabelo, realizar a paramentação com uso de touca e máscara, higienizar as mãos, massagear as mamas com movimentos circulares, limpar as mamas com auxílio de gazes e água limpa ou soro fisiológico e iniciar a coleta após desprezo dos primeiros jatos e coletar o LH em frasco de vidro com tampa de plástico esterilizado. Em caso de uso de bomba extratora, a esterilização dos utensílios também é recomendada^(10,11)^. Imediatamente após a extração, o LH deve ser rotulado e armazenado em estado de congelamento a -20^o^C, em freezer ou congelador, na tentativa de preservar os nutrientes e evitar a proliferação de microrganismos patogênicos^(13)^.

Quanto à distribuição, o Brasil determina que o LH só deve ser administrado cru, ou seja, sem passar pelo processo de pasteurização, quando extraído à beira leito e/ou no PCLH de mãe para filho, sob a supervisão de um profissional de saúde^(13)^. Por outro lado, o LH coletado no domicílio deve, necessariamente, passar pelo controle de qualidade e, em seguida, ser pasteurizado antes de sua distribuição, independentemente de ser LH de mãe para filho^(10,14)^, uma vez que possui risco de contaminação secundária durante a extração, o que compromete a qualidade e segurança do LH a ser oferecido^(10,14,15)^.

Apesar desta recomendação no Brasil, outros países permitem a oferta de LH cru de mãe para filho, independentemente do local de extração^(16)^. Os estudos que comparam riscos e benefícios entre os locais de extração de LH no tocante à qualidade higiênico-sanitária são escassos^(17,18)^. Um estudo realizado em 2014 na Austrália comparou amostras de LH coletados no BLH e em domicílio, e não identificou diferença significativa quanto ao local da coleta do LH, apresentando uma taxa de aprovação de 98% e 94% das amostras coletadas no banco de leite e em domicílio, respectivamente^(19)^.

Desta forma, a análise da qualidade higiênico-sanitária do LH coletado em domicílio, bem como a sua comparação com o LH coletado no PCLH, poderá embasar as práticas já estabelecidas e contribuir para atualização das normas atuais.

Neste sentido, o presente estudo tem como objetivo analisar os fatores associados à qualidade higiênico-sanitária do leite humano doado quanto ao perfil da doadora e local da extração.

## MÉTODO

### Desenho e Local do Estudo

Trata-se de um estudo transversal com coleta de dados retrospectiva de registros de amostras de leite humano doadas para o Banco de Leite Humano de um Hospital Universitário, que integrou o projeto intitulado “Avaliação dos indicadores de um Banco de Leite Humano”.

O BLH da Universidade Federal de São Paulo (UNIFESP) está localizado na região Sudeste do Brasil e realiza cerca de 800 visitas domiciliares/ano para a coleta de LH, obtendo aproximadamente 600 litros/ano. O PCLH distribui cerca de 90 litros de LH/ano para uma média de 260 crianças/ano. A maioria das crianças que recebem o LH doado é prematura internada na UTI Neonatal do Hospital Universitário/Hospital São Paulo (HU/HSP).

### População e Critérios de Seleção

A amostra foi composta por todos os registros de doação do LH no período de 2014 a 2019. Foram incluídos os registros de LH de doadoras consideradas aptas para a doação, de acordo com os critérios estabelecidos pela Rede Brasileira de Bancos de Leite Humano e Agência Nacional de Vigilância Sanitária (ANVISA)^(10)^. O critério de exclusão estabelecido foi o registro de LH doado com dados incompletos relacionados à data de nascimento das doadoras e das crianças, que impossibilitam a análise do leite doado e, consequentemente, impedem de responder ao objetivo proposto no estudo.

### Coleta de Dados

A coleta de dados foi realizada por duas pesquisadoras treinadas para a obtenção das variáveis selecionadas para o estudo no prontuário eletrônico das doadoras e na planilha de dados de produção mensal do BLH, no período de agosto de 2019 a agosto de 2020.

Foram coletadas variáveis para a caracterização da doadora: idade materna (≤ 29 e > 30 anos; subdivisão de faixa reprodutiva do Instituto Brasileiro de Geografia e Estatística - IBGE), período pós-parto da doação (≤1 mês e >1 mês); e variáveis relacionadas ao LH doado: fase do leite humano (colostro, transição e maduro) e valor energético do leite humano (hipocalórico: < 500 kcal/l, normocalórico: 500–700 kcal/l e hipercalórico: > 700 kcal/l). As variáveis de desfecho estabelecidas foram: tipo de doação (exclusiva ou não-exclusiva), local da extração (PCLH e domicílio) e qualidade higiênico-sanitária do LH doado. A qualidade higiênico-sanitária foi verificada pela: 1) presença de sujidade, 2) *off flavor*, 3) acidez Dornic e 4) análise microbiológica. Para tanto, considerou-se qualidade comprometida quando havia presença de sujidade, odor alterado, acidez alterada e contaminação microbiológica^(10)^.

A sujidade foi definida com base em asseio pessoal (presença de pelos, cabelos e pele dentro do frasco de leite) e/ou ambiente (presença de fiapos de tecido, insetos e alimentos dentro do frasco de leite), sendo categorizada de acordo com sua origem.

O teste *off flavor* foi determinado pela avaliação do odor, previamente ao processo de pasteurização, em que a presença de cheiro de sabão de coco, peixe, remédio, cloro, plástico e/ou borracha caracterizam o leite como inapropriado para consumo.

A determinação da acidez no leite humano foi medida pela Acidez Dornic (AD), na qual o valor ≤ 8°D é considerado adequado, utilizando-se um acidímetro de Dornic com ponta fina, graduado de 0,01 mL, uma solução-padrão de hidróxido de sódio 0,1N fatorada e uma solução indicadora de fenolftaleína hidroalcoólica a 1% em álcool 95°GL (noventa e cinco graus Gay-Lussac) neutralizada.

A análise microbiológica foi feita pela inoculação de quatro alíquotas de 1 mL cada de LH pasteurizado, pipetadas de forma independente e inseridas em tubos com 10 mL de caldo bile verde brilhante (BGBL), a 50 g/L (5% p/v), com tubos de Durham em seu interior. Após a inoculação e incubação a 36 ± 1°C, a presença de gás no interior do tubo de Durham caracteriza resultado positivo, sendo o parâmetro aceitável negativo ou ausente.

Para a comparação dos tipos de doação, consideraram-se os dados das mulheres que se manifestaram voluntariamente para doar seu LH, chamadas de doadoras (ou doação) não-exclusivas, enquanto aquelas com filhos internados foram denominadas de doadoras exclusivas. Quanto ao local de extração do LH doado, uma vez que as doadoras exclusivas extraíram seus leites tanto no PCLH quanto no domicílio, estes dois locais foram considerados na comparação da qualidade higiênico-sanitária do LH entre as doadoras.

O LH doado ao BLH da UNIFESP é transportado congelado, sob cadeia de frio, em recipiente isotérmico com gelo reciclável, mantendo a temperatura máxima de -1^o^C, conforme as recomendações da rede de Bancos de Leite Humano (rBLH)^(7)^. O transporte é realizado por um profissional de saúde treinado. No caso de doadoras exclusivas, o LH é transportado pela puérpera ou por um familiar, após receber instruções para o transporte adequado, que inclui manter o LH congelado em um recipiente apropriado. Ao chegar na instituição, todo LH recebido é verificado para garantir que esteja em conformidade para o processamento, o que inclui verificar se o frasco é adequado, se está dentro da validade e se está congelado corretamente. Se o LH entregue pela puérpera apresentar inadequações, será descartado na recepção.

As informações foram inseridas em um banco de dados construído no Microsoft Office Excel 2016®. Foi realizada a dupla checagem dos registros para confirmação de todas as informações obtidas.

### Análise dos Dados

Na descrição dos dados utilizaram-se variáveis qualitativas e suas respectivas frequências relativas (%) e absolutas (n) e, para as variáveis quantitativas, utilizaram-se média e desvio padrão (DP). Para comparar as variáveis, tipo de doadoras, local de extração e qualidade higiênico-sanitária do LH foi utilizado o teste qui-quadrado ou exato de Fisher, considerando o nível de significância estatística α igual a 5% (p < 0,05). Para análise dos dados foi utilizado o software Stata/SE 14.0 (Texas, EUA).

### Aspectos Éticos

O estudo foi aprovado pelo Comitê de Ética em Pesquisa da Universidade Federal de São Paulo (projeto n. 0712/2018, parecer n. 2.755.431), conforme resolução 466 de 2012 da CONEP. Por se tratar de um estudo retrospectivo, utilizando dados de prontuário, o uso do Termo de Consentimento Livre e Esclarecido foi dispensado.

## RESULTADOS

Um total de 14.824 amostras de LH provenientes de 986 doadoras foi analisado. A maioria das nutrizes apresentava mais de 30 anos (75,2%; 30,7 anos; DP = 6,6) e, no momento da doação de LH, encontrava-se com mais de um mês pós-parto (77,7%). As amostras de LH doadas foram, em sua maioria, classificadas como maduras (89,2%) e normocalóricas (54,2%). A maioria das amostras era de doadora não-exclusiva (66,4%), com predominância do domicílio como local de extração (77,3%) (Tabela 1).

**Tabela 1:** Caracterização das doadoras e das amostras de leite humano extraídas, segundo o tipo da doadora – São Paulo, SP, Brasil, 2014–2019.

Variáveis de caracterização	Doadoras não-exclusivas n (%)	Doadoras exclusivas n (%)	p
**Doadoras**			
*Idade da nutriz* (n = 986)			0,867
≤ 29 anos	2.441 (24,8)	1.229 (24,7)	
> 30 anos	7.402 (75,2)	3.752 (75,3)	
*Período pós-parto* (n = 14.824)			0,818
≤ 1 mês	2.198 (22,3)	1.104 (22,2)	
> 1 mês	7645 (77,7)	3.877 (77,8)	
**Doação de LH**			
*Local da extração* (n = 14.824)			**< 0,001**
Domicílio	9.843 (100)	1.617 (32,5)	
PCLH	0 (0)	3.364 (67,5)	
*Fase do leite* (n = 14.816)			**< 0,001**
Colostro	6 (0,1)	137 (2,8)	
Transição	191 (1,9)	1.271 (25,5)	
Maduro	9.646 (98,0)	3.565 (71,7)	
*Valor calórico* (n = 11.210)			**0,001**
Hipocalórico	1.734 (23,5)	868 (22,7)	
Normocalórico	3.905 (52,9)	2.162 (56,4)	
Hipercalórico	1.739 (23,6)	802 (20,9)	

A presença de sujidade foi encontrada em 22% (n = 1.095/4.981) das amostras de LH de doação exclusiva, enquanto de doação não-exclusiva esse número foi de 25% (n = 2.459/9.843), sem diferença estatisticamente significante. E quando avaliada pelo tipo, a sujidade classificada como ambiental foi mais prevalente nas amostras de doação não exclusivas (p = 0,039). No que se refere ao teste *off flavor,* a minoria, 0,1% (n = 18/14.824), das amostras de LH apresentou alterações, sendo que aquelas com odores alterados foram extraídas em domicílio, por doadoras não-exclusivas (p < 0,001). Em relação ao exame de titulação de acidez (AD), a maioria dos LH, 98,9% (n = 12.868/13.018), estava adequada. Acerca da análise microbiológica, dentre as amostras alteradas, 76,3% (n = 748/981) foram de doadoras não-exclusivas (p < 0,001).

Na Tabela 2 observa-se que, dentre os fatores associados às alterações higiênico-sanitárias do LH extraído, ter mais de 1 mês pós-parto e ter realizado a extração em domicílio estiveram relacionados com a presença de sujidade (p < 0,001 e p = 0,048, respectivamente). Além disso, a doação não-exclusiva (p < 0,001) de LH maduro um mês pós-parto (p = 0,024) e a extração do LH em domicílio (p < 0,001) estiveram relacionadas com alteração da acidez. A doação não-exclusiva, especialmente entre mulheres com mais de 30 anos de idade e aquelas que realizaram a extração em domicílio, esteve associada à alteração do resultado da análise microbiológica (p < 0,001, p = 0,004 e p < 0,001, respectivamente).

**Tabela 2:** Fatores associados ao controle higiênico-sanitário de leite humano extraído – São Paulo, SP, Brasil, 2014–2019.

	Sujidade			Off flavor			Acidez			Análise microbiológica*		
	Ausência n (%)	Presença n (%)	p	Normal n (%)	Alterado n (%)	p	Normal n (%)	Alterada n (%)	p	Normal n (%)	Alterada n (%)	p
**Característica da doadora**
Não-exclusiva	7.584 (66,1)	2.259 (67,4)		9.829 (66,4)	14 (77,8)		8.462 (65,8)	137 (91,3)		6.691 (64,9)	748 (76,3)	
Exclusiva	3.886 (33,9)	1.095 (32,6)	0,184	4.977 (33,6)	4 (22,2)	0,306	4.406 (34,2)	13 (8,7)	**<0,001**	3.617 (35,1)	233 (23,7)	**<0,001**
**Idade da nutriz**
≤ 29 anos	2.866 (25)	804 (23,9)		3.666 (24,8)	4 (22,2)		3.180 (24,7)	40 (26,7)		2.537 (24,6)	282 (28,7)	
> 30 anos	8.604 (75)	2.550 (76)	0,231	11.140 (75,2)	14 (77,8)	0,803	9.688 (75,3)	110 (73,3)	0,581	7.771 (75,4)	699 (71,3)	**0,004**
**Período pós-parto**
≤ 1 mês	2.539 (22,1)	763 (22,8)		3.299 (22,3)	3 (16,7)		2.869 (22,3)	45 (30)		2.271 (22)	222 (22,6)	
> 1 mês	8.931 (77,9)	2.591 (77,2)	0,453	11.507 (77,7)	15 (83,3)	0,567	9.999 (77,7)	105 (70)	**0,024**	8.037 (78)	759 (77,4)	0,666
**Local de extração**
Domicílio	8.713 (76,0)	2.747 (81,9)		11.444 (77,3)	16 (88,9)		9.812 (76,3)	142 (94,7)		7.765 (75,3)	805 (82,1)	
PCLH	2.757 (24,0)	607 (18,1)	**<0,001**	3.362 (22,7)	2 (11,1)	0,240	3.056 (23,7)	8 (5,3)	**<0,001**	2.543 (24,7)	176 (17,9)	**<0,001**

*n = 11.289.

## DISCUSSÃO

A extração de LH no domicílio, principalmente por doadoras não-exclusivas com mais de 30 anos e no período de 30 dias ou mais pós-parto, mostrou-se associada a maior prevalência de não conformidade do LH, relacionada a insuficiência de cuidado higiênico-sanitário no momento da extração do leite devido à presença de sujidade, aumento da acidez e alteração de fatores microbiológicos no leite doado. Este é o primeiro estudo que demonstra os possíveis riscos do uso de LH cru coletado em domicílio, reforçando a importância da pasteurização.

Apesar da ampla recomendação do uso do LH cru de mãe para filho, dada sua importância e benefícios imunológicos^(2)^, há necessidade de garantir a segurança alimentar do LH oferecido ao recém-nascido prematuro^(20)^, visto suas características e taxa de mortalidade^(21)^. O presente estudo mostrou que o LH extraído no domicílio apresenta maior taxa de sujidade (p < 0,001), o que vai ao encontro da recomendação da rBLH de analisar a qualidade de todos os LHs extraídos em domicílio^(20)^.

A contaminação por sujidade ocorre principalmente devido ao contato com o meio externo e está frequentemente associada a descuidos durante a técnica de extração como: falta de higiene das mãos e das mamas, o uso de cabelos soltos, a manipulação de objetos enquanto realiza a extração, a exposição do recipiente ao ambiente e a esterilização inadequada do recipiente^(10,22)^. No presente estudo, observou-se que a maioria das amostras contaminadas com sujidade estava relacionada aos cuidados com a higiene pessoal e com o ambiente durante a coleta. Um estudo que avaliou o impacto de medidas adotadas para reduzir a quantidade de LH impróprio, por meio de instrumento educativo com o passo-a-passo da técnica de extração láctea e as causas de descarte do LH, mostrou uma redução da sujidade de 24% para 10,5%, reforçando a importância da orientação constante com o intuito de manter a qualidade no decorrer do tempo^(23)^.

Além do risco de contaminação por sujidades durante a extração, o LH apresenta grande capacidade de absorção de substâncias voláteis. A detecção da característica sensorial não-conforme, quando comparada ao aroma original do LH, por meio da percepção do olfato utilizado como forma de classificação do LH como apropriado ou não ao consumo, é um método rápido e eficiente^(10)^. No presente estudo, a taxa de alteração do odor foi identificada apenas entre amostras que foram extraídas em domicílio e por doadoras não-exclusivas, apesar de mínima. Um estudo observou relação direta entre a modificação de odor e a contaminação microbiológica do LH em razão da manipulação e/ou estocagem inadequada, como a contaminação do LH por microrganismos lipolíticos associados a odor de sabão de coco^(24)^. A recomendação para a extração de LH em ambiente livre de odores, tais como perfume e temperos, garante a qualidade do produto e reforça a necessidade de proteção do LH extraído^(24)^.

Outro parâmetro que classifica e seleciona o LH é a acidez Dornic. A baixa taxa de alteração da acidez no LH (1,1%) encontrada no presente estudo corrobora o estudo realizado em Uberlândia^(25)^. Por outro lado, quando comparado com outra pesquisa realizada na Espanha, que identificou uma AD de 12% no LH^(26)^, a prevalência na alteração de acidez superior aos valores recomendados foi significativamente maior quando a extração foi realizada no domicílio (p < 0,001). A acidez original do LH varia entre 1,0 e 4,0°D e, em ambiente favorável ao crescimento biológico, a acidez aumenta pela produção de ácido. Este processo de elevação da acidez no LH, quase sempre, está associado a ações de microrganismos, o que reduz o valor nutricional e imunológico e torna o LH impróprio para o consumo^(13)^. Além disso, outros fatores que influenciam a variação da acidez são: manipulação inadequada do LH, armazenamento em recipiente impróprio ou esterilizado de forma incorreta e oscilação de temperatura, seja pela demora em armazenar o LH ou problemas relacionados ao refrigerador^(10)^.

Estes achados demonstram a importância da orientação e monitoramento constantes da doadora de LH, para reduzir o recebimento de LH doado impróprio para consumo^(27)^. Destaca-se a importância de reduzir a doação de LH com acidez alterada por meio da orientação efetiva das doadoras quanto aos cuidados com o manuseio dos recipientes esterilizados disponibilizados para a coleta do LH, do congelamento imediato à extração e manipulação mínima após o armazenamento, bem como correto transporte até o BLH^(10)^.

Quanto à análise microbiológica, este é o último parâmetro de qualidade higiênico-sanitária a ser avaliado, que identifica retenção de patógenos por excesso ou falha de manipulação. Este é realizado após o tratamento térmico do LH doado e objetiva inativar microrganismos como *Staphylococcus aureus, S. epidermidis, Enterobacteriaceae, fungos e leveduras* que podem ser encontrados no LH^(10,22,28)^. A taxa de contaminação microbiológica de 8,7% foi similar ao estudo realizado em um BLH norte-americano^(28)^. A contaminação identificada no presente estudo foi maior entre mulheres acima de 30 anos (p = 0,004), em doação de LH não-exclusiva (p < 0,001) e que foi extraída no domicílio (p < 0,001), diferentemente do estudo realizado na Espanha, em que a contaminação foi maior entre doadoras exclusivas^(29)^. Estes achados reforçam a necessidade de controle rigoroso de qualidade do LH doado extraído em domicílio^(30)^, bem como o preparo adequado das nutrizes que desejam realizar a doação.

Dentre as limitações deste estudo, o uso de dados retrospectivos e uma análise de recorte transversal podem limitar a inferência dos resultados, além do menor número de doações exclusivas em comparação às não-exclusivas. Por outro lado, os achados mostram-se importantes para fortalecer as recomendações atuais da rBLH quanto à importância do controle de qualidade do LH doado.

## CONCLUSÃO

O comprometimento da qualidade higiênico-sanitária do LH doado deu-se especialmente entre doações não-exclusivas e quando realizadas no domicílio.

O achado dá suporte à recomendação quanto ao processamento do LH de doadoras exclusivas, quando extraído em domicílio, e reforça a importância de as equipes de saúde que atuam em BLH e PCLH estabelecerem uma rotina de orientações individualizadas para melhor aproveitamento do LH doado, a fim de garantir a qualidade higiênico-sanitária do LH distribuído aos prematuros.
